# Upper Limb Therapy for Infants and Young Children with Unilateral Cerebral Palsy: A Clinical Framework

**DOI:** 10.3390/jcm13226873

**Published:** 2024-11-15

**Authors:** Susan Greaves, Brian Hoare

**Affiliations:** 1 CPGroup, 74 Faraday Street, Carlton, VIC 3053, Australia; brianhoare@cpteaching.com; 2 School of Allied Health, Australian Catholic University, 115 Victoria Parade, Fitzroy, VIC 3065, Australia; 3 Discipline of Occupational Therapy, La Trobe University, Plenty Road, Bundoora, VIC 3086, Australia

**Keywords:** unilateral cerebral palsy, upper limb, early intervention, bimanual therapy, constraint-induced movement therapy

## Abstract

Early detection and rehabilitation interventions are essential to optimise motor function in infants and young children with unilateral cerebral palsy. In this paper we report a clinical framework aimed at enhancing upper limb therapy for infants and young children with unilateral cerebral palsy during a sensitive period of brain development. We describe two major therapeutic approaches based on motor learning principles and evidence: constraint-induced movement therapy and bimanual therapy. These two therapies have demonstrated efficacy in older children and emerging evidence is available for their application to infants younger than 2 years of age. To provide clinicians with guidance as to when to implement these therapies, we discuss the key consideration when undertaking upper limb therapy programs. In addition, we describe the factors to consider when choosing which approach may be suitable for an individual child and family. Detailed strategies for implementing these therapies in infants and young children of different ability levels are given.

## 1. Introduction

Children with unilateral cerebral palsy (unilateral CP) have an impairment that primarily affects one side of their body. The early motor impairment can involve spasticity, dystonia or a combination of both, and presents with varying degrees of abnormal posturing of the arm/hand into shoulder internal rotation, elbow flexion, forearm pronation and fisting of the fingers with the thumb adducted/flexed and across the palm [1]. These postural changes cause difficulty using the more-affected upper limb to perform both unimanual and bimanual actions. For infants with unilateral CP early asymmetrical hand use and persistent fisting of the more-affected hand are often the first signs of concern [2]. Compensatory behaviours and limb disuse during early play experiences limit opportunities for children with unilateral CP to learn new skills and strategies and lead to aberrant neuroplastic changes [1,3].

In recent years there have been considerable efforts to detect and diagnose infants with CP as early as possible [4,5,6]. This is to ensure that therapy can commence immediately to optimise the sensitive period of brain development [7] when infants are most rapidly learning actions and behaviours [8]. The best available evidence supports therapy models based on contemporary motor learning principles [9,10]. Two upper limb specific models of therapy based on motor learning include constraint induced movement therapy (CIMT) and bimanual therapy. Both have been shown to be effective for children older than 2 years of age [11,12] and there is a growing body of research supporting these models for infants with CP [13,14,15,16,17,18,19,20,21,22,23].

Evidence for CIMT and bimanual therapy has primarily been established using randomised controlled trial (RCT) methodologies where children are randomly allocated to receive either therapy. In clinical settings, however, a decision to use these models of therapy is more nuanced and requires consideration of a number of child and family factors which can contribute to the success of the therapy program. Guided by extensive clinical and research expertise in the field, the aim of this paper is to assist clinicians to implement evidence-based upper limb therapy for infants with unilateral CP under two years of age in clinical practice. We will briefly outline each therapy before discussing the key considerations when undertaking upper limb therapy programs. We will also describe the factors to consider when choosing which approach may be suitable for an individual child and family as well as providing detailed strategies for implementing these therapies in young infants of different ability levels.

## 2. Upper Limb Therapies for Infants with Unilateral CP

For children older than 2 years of age, there are many different approaches of CIMT and bimanual therapy that have been described in the literature [11,24], with a broad variation in the dosage, content, and mode of delivery [10]. Both CIMT and bimanual therapy have been applied using an *intensive* or *distributed* practice approach. *Intensive* approaches are implemented using high duration and frequency of sessions over a short period, such as 6 h per day, 5 times per week for 2 weeks. Whereas *distributed* approaches are implemented using lower duration and frequency of session over a longer period (for example, 2 h per day, 5 times per week for 8 weeks) to optimise learning outcomes. It is important to recognise that both *intensive* and *distributed* approaches to therapy have shown to be effective for children with unilateral CP older than 2 years of age [9,11,12], and there is an increasing number of clinical trials using these approaches for infants with CP under two years of age [13,14,15,16,17,18,19,20,21,22,23].

### 2.1. Constraint Induced Movement Therapy

Constraint induced movement therapy (CIMT) provides an opportunity for repetitive practice of unimanual movements and actions [25]. The two key components of CIMT include restraint of the less-affected upper limb (irrespective of what constraint is used) and intensive, structured practice (irrespective of how this is done) [26]. The intensive practice aims to drive neuroplastic changes, mitigate secondary musculoskeletal impairments and improve function [25]. The original *intensive* model of signature CIMT developed by Edward Taub [27] was first modified by Eliasson et al. in 2005 for children aged 18 months to 4 years (mCIMT) [28]. This was the first *distributed* approach of mCIMT. More recently, Eliasson modified the mCIMT protocol for use with infants which is now known as Baby-CIMT [13].

The aim of Baby-CIMT is to increase the amount and quality of hand use in the more-affected arm and hand. It is informed by dynamic systems theory, which places emphasis on self-initiated motor actions which is crucial for motor development. Baby-CIMT uses a simple soft restraint such as a sock or bag clip at the end of a sweater to block use of the less-affected hand to encourage use of the more-affected hand. It is implemented in two, 6-week blocks of therapy separated by a 6 week pause. The *distributed* practice approach involved 30 min of practice 6 days per week for a total dosage of 36 h [13]. Similar models of CIMT designed for young infants have also used a *distributed* practice approach, although with some variation in the amount of time per day, the number of days per week and the number of weeks it is implemented [16,17,18]. It has also been implemented using a telerehabilitation model [15].

### 2.2. Bimanual Therapy

Hoare and Greaves (2017) define bimanual therapy as “a process of learning bimanual hand skills through the repetitive use of carefully chosen goal related, two-handed activities that provoke specific bimanual actions and behaviours” [25] (p. 52). While less well-described in the literature for infants under 2 years of age, bimanual therapy is grounded in concepts from cognitive, perceptual, and motor-based theoretical frameworks and aims to provide opportunities for children with unilateral CP to practice and learn bimanual skills and strategies that are targeted to their individual ability level [16,19]. Bimanual therapy for infants has primarily been implemented using *distributed* practice approaches that vary in dosage [16,17,18,19]. An *intensive* approach of bimanual therapy developed by Charles and Gordon in 2006 is Hand Arm Bimanual Intensive Therapy (HABIT) [29]. HABIT, guided by shaping theory, focuses on structured massed practice that increases in complexity using functional activities that necessitate bimanual hand use [29]. While HABIT has not been evaluated in infants less than 2 years of age, an adapted protocol (HABIT-ILE) that concomitantly includes a focus on the lower extremities and sustaining postural control as well as bimanual hand use [30] has been used with young infants aged 6–18 months [20]. This was implemented in a camp setting where therapy was provided for 5 h per day, 5 days per week for 2 weeks (total 50 h).

### 2.3. Hybrid Therapy

In addition to providing either CIMT or bimanual therapy, upper limb therapy programs can include both therapies, either in the same session, or immediately after unimanual training [26]. Hybrid models of upper limb therapy were developed to improve outcomes of CIMT, with the key ingredients of CIMT always included and bimanual training added to different extents [26]. In infants with unilateral CP, the combination of therapies has been used in an *intensive* approach providing CIMT (6 h a day for 28 days) with less intensity of bimanual therapy (20 min a day) [21]. *Distributed* hybrid approaches of roughly equal CIMT and bimanual therapy duration have also been described, with some variation in the amount of time per day (30–60 min), the number of days per week (5–7) and the number of weeks it was implemented (8–10) [18,23].

While there is growing research evidence supporting motor learning-based upper limb therapy models in infants and young children with unilateral CP, information about how to support implementation in clinical practice has not yet been described. As with children older than 2 years of age, considerations for which therapy approach might be most suitable for an individual child and their family would help clinicians select the most appropriate therapy for a given situation [31]. The next section provides a summary of key considerations when undertaking evidence-based upper limb therapy programs for infants with CP less than 2 years of age.

## 3. Considerations for the Implementation of Evidence-Based Upper Limb Therapies for Infants and Young Children

Therapy for children with CP, or at high risk of CP, should be provided as early as possible, be based on motor learning theory and adhere to key principles for driving activity-dependent plasticity to optimise brain development. These principles include specificity, salience and adequate dosage of practice. Knowledge about the development of hand skills, perception and cognition as well as the importance of play for infants is also critical to help support families in understanding when skills develop and to guide the collaborative goal setting process. This section of the paper provides a summary of these key considerations.

### 3.1. What Is Motor Learning Theory?

Motor learning theory recognises the complex and interactive processes of acquiring and generalising skills through practice and experience [32]. Contemporary motor learning theory is informed by several theories [33,34,35,36] that guide our understanding about how humans acquire, generalise, and transfer new skills. Operationalising multiple theories into a discrete therapy approach is complex, but it is critical [10]. Clearly defining the discrete strategies used in a therapy approach is essential for guidance about how to implement the specific approach in clinical practice. These strategies are the instructions for what to do, and when to do it. Reporting specific strategies, based on guiding theories, also allows researchers to articulate the differences between various therapy approaches [10].

In the 1950s, the Bobaths provided explicit detail about a therapy approach [37] that was based on theories of motor recovery developed in the late 1800s and early 1900s [38]. The approach was very clearly defined and significant infrastructure was developed to support the teaching and training of this specific approach. As a result, the Bobath approach, also known as Neurodevelopmental Therapy (NDT) went on to become the dominant approach used worldwide for the treatment of children with CP [39]. This was despite antithetical views at the time that promoted interventions such as strength training [40]. Despite its dominance in clinical practice, a recent systematic review called for the de-implementation of NDT in clinical practice due to evidence demonstrating no difference in effect between NDT and control, or between different doses of NDT. A strong recommendation was made for the use of motor learning-based models of therapy [39].

In contemporary motor learning-based therapy approaches, the environment is used to motivate and provoke a child to use spontaneous and purposeful actions. These interactions provide experiences that guide future actions and increase proficiency. They lead to a cascade of development across all domains including motor, sensory, cognitive, perceptual, etc. The aim of a motor learning-based therapy approach is not to normalise movements [41], but to consider the interaction between the child, the task and environment, and to support infants and children in learning how to achieve success and independence in whatever ways they can. The task outcome is most important, rather than how it is done, because the more they do, the more they learn.

Most contemporary activity-based therapy approaches are primarily based on dynamic systems theory [35,36]. Pioneers such as Anne Gentile (1992) and Kaplan and Bedell (1999) were early proponents, and introduced a model of skill acquisition for children with movement disorders [42,43]. This saw the development and introduction of approaches such as CIMT and bimanual therapy into the field of upper limb CP research in the early 2000s, resulting in a shift away from facilitative therapy approaches [37]. However, this shift in research has not necessarily been accompanied by equivalent resources required to support therapists in the implementation of motor learning therapy approaches in clinical practice [10]. Inadequate reporting across 103 evidence-based and task-focused upper limb therapy approaches has been unhelpful and has had a significant impact on the implementation of contemporary models of therapy [10,44]. Motor learning theory does not just represent a change in technique but a change in basic thinking [42] when compared with theories used to inform models of therapy such as the Bobath approach [37]. As a result, the strategies applied are not at all reconcilable. For example, no CIMT or bimanual therapy approach aims to inhibit tone or primitive reflexes, use weightbearing activities, or facilitate movement from the start to finish of the movement/action. In motor learning approaches, a therapist’s hands can be used, but only to show the child how to obtain a reference for the movement/action. A therapist then provides support and motivation for the child to spontaneously and repetitively initiate the movement/action. Changes to the environment and the task are used to increase or decrease the challenge and to provoke the goal-related movements/actions. Considerations about the type of task, type of practice, and type of feedback are essential in the application of motor learning approaches [10].

### 3.2. Goal Setting to Ensure Specificity of Practice

Implementation of evidence-based upper limb therapy does not simply include playing with a child or providing toys in the hope that they will learn something from this experience. In fact, many children with unilateral CP will often avoid challenging activities and use persistent behaviours such as throwing [45] or mouthing of toys. These behaviours can often dominate early play behaviours and significantly limit motor, cognitive and perceptual experiences that promote learning and skill development.

Upper limb therapy for infants and young children must be based on specific and explicit action-focused goals. Along with consideration of family factors, these goals guide the clinical reasoning process for the selection of the most appropriate model of therapy. As with all health-related conditions, it is not appropriate to first select a treatment and then consider the goal(s). In children with unilateral CP, to use the hand more or to use the hand better are not specific or explicit. How does a caregiver support a child to achieve these goals? When will a child use their more-affected hand enough and what is better? Explicit action-focused goals are critical for ensuring that the practice is individualised (specific) and outcomes are measurable. In addition, when goals are explicit, e.g., to grasp objects using the assisting hand from the dominant hand, this enables toys to be selected that are matched to these goal-related actions. In a fun and motivating environment, it is a combination of the toys and the strategies used by a therapist during play that optimises skill development. This is consistent with Gentile’s advice that a therapist has two primary functions in promoting early skill acquisition: (1) to create a specific environmental problem (using a toy that provokes a difficult goal related action) and (2) to establish an adequate motivational level of the child to solve the problem [42].

### 3.3. Promoting a Salient and Moderately Challenging Experience

Learning requires motivation and attention [46,47]. Therefore, consideration of motivational factors that will drive an infant or young child to want to play with a toy need to be carefully considered in upper limb therapy approaches. To motivate an infant or young child and to ensure attention to their environment, the practice experience needs to be sufficiently salient, such as a set of reasons which draw an observer’s attention toward a particular object [48]. There is a neural system that mediates saliency, and engaging this system is critical for driving experience-dependent plasticity [48]. Research in the adult literature demonstrates that long-term plasticity is dependent on goal-directed, task-specific, and challenging practice, not mere repetition of simple movements [49]. Upper limb therapy approaches should therefore be embedded in goal-related play that provides opportunities for enjoyable experimentation and repeated practice adopting different strategies to find the optimal solutions and to develop skilled performance [50].

Therapy sessions should not only be fun, enjoyable and have meaning, they should also provide a challenge. Practice should involve repeated attempts to produce motor behaviours that are beyond the present capability [51]. It is not simply repeating movements and actions that are already part of a child’s motor repertoire. In animal models, repetition of previously acquired motor movements has been found to not result in significant synapse addition or map expansion in the motor cortex [52]. When a child is provided with a task that is challenging (i.e., possible at the child’s upper limit of capabilities or zone of proximal development) and motivating, it is more likely that motor skill acquisition will be improved [43,53]. Learning takes place when the ability of the child and the difficulty of the task are well-matched.

### 3.4. Importance of Dosage of Practice

Physical practice is an important determinant factor of motor skill acquisition [33,34]. Motor learning-based therapy approaches recognise that repetition of practice is a fundamental strategy for achieving skill acquisition, transference and generalisation. It is repetition of practice that allows an infant or young child to progress from the cognitive and associative phases of learning to an autonomous phase [54]. However, the amount of practice or dosage of practice is only one strategy that operationalises motor learning theory. Motor learning-based therapy approaches also contain other strategies of equal importance. As stated in Taghizadeh et al. (2022), the dosage requirements that inform a specific approach is guided by the theories that underpin the approach [10]. We have previously outlined the fundamental differences between CIMT and bimanual therapy approaches for children with unilateral CP [25]. How a therapist manipulates the environment and varies the type of task, and the type of feedback will also greatly influence a child’s learning and generalisation of skills.

### 3.5. Home Programs and Supporting Families

Home programs are “therapeutic activities that the child performs with parental assistance in the home environment with the goal of achieving desired health outcomes” [55] (p. 463). There is high-level evidence supporting home programs for children with CP [56,57]. They are an integral part of every *distributed* motor learning-based upper limb therapy approach as they provide a pragmatic solution to achieving the required dose of practice for skill acquisition and transference [57]. Home programs are only effective if the program content is designed upon proven effective interventions [57]. As a result, the structure and focus of home programs have dramatically changed over the last few decades. Guided by knowledge from motor learning theory and the principles for activity dependent plasticity, there has been a move away from encouraging families to do “work”, “exercises”, or passive stretches with their child [58].

Effective home programs require that they be set up in partnership with the parent considering the family’s preferences and individual situation [57]. Parents should be acknlowledged as the expert in knowing their child and their home situation. The therapist and caregiver should collaborate to set realistic and appropriately time-framed goals for their child. The home program should be established by choosing the best evidence-based intervention to match the desired goals and family situation and caregivers should be provided with education about the key ingredients of the therapy approach. Coaching should be provided about how to enrich their child’s environment to provoke as many opportunities as possible to practice the desired goals during daily routines, as well as empowering parents to devise or exchange the activities to match their child’s preferences and their own individual environment. Guidance should also be provided about how to position their child; how to provoke goal-related, self-generated movements in the context of play; how to use motor learning strategies and the environment to grade complexity of the task to optimise skill acquisition; and how to match toys to provoke goal related actions. To evaluate the outcomes of the home program, regular support and coaching should be provided to identify a child’s improvement, collaboratively adjust the home program and evaluate outcomes together [45,57,59].

From a broader perspective, support should be provided to caregivers to instil feelings of hope, self-confidence, and motivation about implementing home programs [60]. Parents’ capacities to optimally support infants and children to attempt and persevere with potentially challenging tasks should be considered a core aspect of evidence-based therapy programs [60]. Sustained, goal-directed persistence on moderately challenging tasks is considered a key indicator of mastery motivation [61].

### 3.6. Development of Hand Skills

The focus of contemporary upper limb therapy for infants and young children with unilateral CP is not to achieve “typical” or “normal” patterns of movement. These approaches aim to support children in learning how and when they can use specific upper limb actions and skills to independently and successfully perform activities to meaningfully interact with their environment. In early life, the context for these learning experiences is in play—the primary occupation for infants and young children.

However, while we do not expect infants and young children with unilateral CP to use their arms/hands like typically developing children, due to the rapid nature of development in the first two years of life, knowledge about what to expect children to be able to do when they use their hands to interact with toys and objects in their environment is critical. It is essential to know what is possible and what is not possible [62]. For example, at what age can you expect infants to be able to spontaneously reach for objects [63] or be able to use different types of grasps for different objects [64]? Or, when can infants use each hand independently from the other so that one hand can be used as a stabilising/holding hand while the other manipulates in role differentiated bimanual manipulations [65]? Attempting to provoke these skills when an infant with unilateral CP is not developmentally ready for these actions is not appropriate [66].

It is only recently that the literature has described how children with unilateral CP develop hand use in the first 12 months of life. The development of the Hand Assessment for Infants (HAI), which is designed for children with neurological concerns, has enabled study of the progression of hand function at an early age. By compiling 489 HAI assessments of typically developing infants between 3 and 10 months of age, Ek et al. (2019) were able to create growth curves that document the development of both unimanual and bimanual actions for typically developing infants [67]. This is an important resource that allows for the interpretation of whether an infant’s HAI results differ from what can be expected. Additionally, for infants less than 12 months of age with unilateral CP, a recent study using the HAI found three distinct trajectories for the development of hand function: a high functioning group who demonstrated a more rapid and continuous acquisition of skills, a moderate functioning group who had slower but steady gains, and a low functioning group who showed a much slower improvement which plateaued at around 9 to 10 months of age [68]. The study authors determined that an important skill for classification to the moderate or high functioning groups was the ability to grasp by 6 months of age. Knowledge gained from this study and the development of the HAI has been critical. It provides an understanding that while infants with a diagnosis of unilateral CP may have the same clinical diagnosis, not all develop unimanual or bimanual actions at the same rate or use the same actions to perform bimanual activities. This supports the need for the tailoring of individualised therapy programs that ensures specificity of practice at the infant/child’s ability level.

Another important consideration for the development of hand skills in infants includes the postural requirements that allow for best use of the hands and learning opportunities. Research has shown that when sitting, as compared to supine and prone, infants aged 5–7 months produce more manual, oral and visual exploration of toys which provides them with increased learning opportunities [69]. For infants with unilateral CP, independent sitting usually develops between 8 and 9 months of age as compared to typically developing infants who usually achieve sitting independence between 6 and 8 months of age [70]. Harbourne et al. (2014) found that increased sitting effort decreases an infant’s ability to visually attend to and detect, extract and process information from their environment [71], as well as interfering with focused attention. Focused attention refers the processes that allow infants to focus on aspects of an object or their environment to enable learning and problem solving [72]. It involves concentrated examination of objects during independent play or object exploration and plays an important role in learning by enhancing selectivity and by maximising the intake and use of information [73]. Developing upper limb skills when exploring and manipulating objects, particularly during the latter half of the first year, facilitates the development of focused attention [74]. As impaired sitting postural control impacts focused attention and provides less opportunity for learning and attending to objects [75], providing adequate support to allow infants with unilateral CP to maintain an upright posture with reduced effort is important for maximising hand use as well as facilitating learning.

### 3.7. Perceptual and Cognitive Skill Development

To improve hand skills in infants and young children with unilateral CP, clinicians require an understanding about how cognition and perception influence hand use and opportunities for learning about their environment. There are a number of theoretical frameworks that emphasise the interrelatedness between cognition, perception, and action in infancy. Embodied cognition states that cognition emerges from bodily interactions within and towards the environment, and that it is inseparable from an infant’s perceptions of the environment and their actions towards it [76,77]. The concept of perception and action being interwoven is also consistent with dynamic systems theory which proposes that behavioural performance emerges from complex interactions between an infant’s abilities, their experiences, and the features of the task and the environment [78,79]. Gibson’s ecological approach views cognition as being inseparable from perception, with perceiving and acting upon those perceptions occurring simultaneously and in continuous cycles [80,81]. Perception is seen as an active process of gaining information about an object or the environment and using this knowledge to perform actions. Depending on the result they may then adapt their actions as they learn about the object they are exploring and the consequences of their actions [82]. Gibson’s theoretical framework also emphasises the importance of affordances to the perception-action cycle. Affordances are the action possibilities that are available to infants when they actively explore an object or their environment [81,83]. The objects that infants explore are seen as being rich in perceptual information that when perceived specifies the type of actions that are used upon it. This requires the infant to be able to attend to, detect, and use the information afforded by the object, but is also influenced by the position of the child, the object itself and the infant’s action capabilities [84,85]. In combination, these theoretical frameworks illustrate that perception-action experiences play an important role in shaping the emergence of upper limb skills and how infants use their hands to play with toys and other objects. It is also the way that infant’s make inferences about the functions and meanings of objects and build knowledge relevant to the task, the object, and the actions required to act upon it. Providing an enriched environment that included parental responsivity and a structured environment with multiple opportunities to explore toys that are specific and appropriately challenging is crucial for the development of perceptual and cognitive skills, and in turn how well an infant can use their hands [59].

### 3.8. Play and Object Affordances

Play is an essential form of exploration for an infant and a major way infants gain knowledge and understanding of the world around them [74,83]. Research suggests that through exploratory play with objects, infant’s visual exploration becomes more efficient, their manual exploration becomes more complex and the link between their visual and motor systems becomes more integrated; all of which lead to increasingly sophisticated cognitive skills and opportunities for learning [86]. However, for children with unilateral CP, early play experiences are significantly altered by delays in postural control, motor difficulties and opportunities for learning about their environment, which in turn impacts the development of play skills. While infants with unilateral CP may experience difficulties playing, as therapists we use play to create the environmental task or challenge that the infant needs to solve by carefully considering the toy properties (or affordances) and the task required to complete the play action. As discussed in the previous section, affordances are the opportunities that an object offers during object exploration. Embedded in the perception-action cycle, an infant perceives the action possibilities of the toy and acts on this possibility, which in turn provides a learning opportunity about the toy and an opportunity for a new perception and new action in future experiences. In upper limb therapy programs careful consideration of toy affordances is fundamental for achieving successful outcomes. It ensures specificity of practice and allows individualisation of therapy programs by matching toys to a child’s specific action-focused goals. Toy affordances include aspects such as the toy’s size, shape or moveable parts. It also includes consideration of presentation of toys to provoke a desired motor response. For example, standing a toy vertically will provoke a different motor response to laying it horizontally on the table [25].

It is important to recognise that the toys themselves will not necessarily provoke repeated practice of goal-related actions. A therapist must also have knowledge about the specific strategies that can be used to motivate an infant/child and understand how to adapt the environment by presenting the toy to provoke the response. These strategies are the observable therapeutic actions that can improve or interfere with learning and generalisation of skills and include the selection and manipulation of tasks, practice and feedback conditions [87]. In addition, other aspects of the toy need to be considered such as whether it is age appropriate, motivating, and fun. These are essential for promoting extended engagement with the toy and repetition of desired manual actions.

## 4. Implementing CIMT or Bimanual Therapy for Infants Under 2 Years of Age: When and Why

Study protocols for RCTs are a useful source of information about the effectiveness of CIMT or bimanual therapy for infants and young children with unilateral CP; however, they do not allow for important considerations that are required for implementing these approaches in clinical practice. This includes the clinical rationale and considerations for when you should use these different therapy models and why. This next section is divided into three parts with the aim of exploring the critical factors that inform the implementation of evidence-based upper limb therapy in clinical practice. It will discuss how an understanding of caregiver’s goals for their child and the family’s unique context, combined with the use of observational-based standardised assessment tools, can be used for setting explicit action-focused goals to ensure specificity of practice and selection of the most appropriate therapy model to achieve these goals.

### 4.1. Collaborative Goal Setting with the Family

Collaborative goal setting with a family is used to identify a child’s strengths, as well as their difficulties, and to establish parental concerns regarding their child’s upper limb performance. It recognises that parents know their child best and can help inform goals that are relevant to their own environment and situations. Collaborative goal setting can also help to decide the type of therapy program that may best be implemented as it also considers the family context and matches the right therapy model to the family and the child. Collaborative goal setting helps to ensure that therapy does not only happen in clinic rooms or when the therapist is present, but that it extends to all settings. This is especially important for distributed models of therapy where home programs are an essential feature of these programs [12,57].

### 4.2. Formulating Action-Focused Goals

Following the collaborative goal setting process the next step is to develop specific action-focused goals that address the parental concerns. To assist this process, naturalistic observations of the child handling toys and other objects can be helpful. However, structured and measurable observation of how a child is using their upper limb(s) using a valid and reliable assessment tool provides significantly greater descriptive information to assist with clinical reasoning and determining specific goals. It also allows for reliable evaluation of change following intervention. The two most commonly used upper limb assessments for infants with unilateral CP are the HAI and the Mini Assisting Hand Assessment (Mini-AHA).

The HAI is a recently developed criterion and norm referenced assessment tool that describes and evaluates upper limb use in infants aged 3–12 months at risk of CP [88]. It uses a semi-standardised, age-appropriate kit of toys to measure active, object-related use of the hands. The HAI comprises 17 items which are each scored on a 3-point rating system. Twelve items are used to measure both the left hand and the right hand, with each hand being scored separately. This allows for the quantification of any differences between the hands through an asymmetry index. A further five items measure how well the infant uses both hands together. Scores from each of the hands as well as the bimanual items are summed together to provide a Both Hands sum score (range 0–88), which is converted to an interval level (0–100) scale called the Both Hands Measure. The HAI has good evidence for validity and reliability [88,89] and also has evidence as a useful tool to help predict whether an infant has unilateral CP or not based on upper limb asymmetry [90].

The Mini-AHA is a valid and reliable, criterion referenced assessment that measures how effectively infants aged 8–18 months with unilateral CP use their more-affected hand during bimanual task performance [91]. It is based on the Assisting Hand Assessment (AHA), which is considered the gold standard assessment for evaluating bimanual performance for children with unilateral CP [92]. Like the HAI, the Mini-AHA uses a semi-standardised set of age-appropriate toys; but in this instance all the toys are designed to provoke bimanual hand use [66]. Twenty items that evaluate how well the more-affected hand is used during bimanual performance are scored on a 4-point rating scale. Scores for the 20 items are added to provide a sum score (0–88), which is then converted to a 0–100 interval level scale (Mini-AHA units) [91].

While these two assessments are both valid and reliable tools for evaluating upper limb function in infants and young children with unilateral CP; when to use either assessment is more nuanced. Firstly, while there are overlapping age periods for when they can be used, the HAI is designed for younger infants (3–12 months), while the Mini-AHA is validated for older children (8–18 months). As well as being a descriptive and evaluative tool, the HAI can also assist with early detection and diagnosis of unilateral CP in infants as young as 4 months [90]. It does this by evaluating the use of both upper limbs in unilateral and bilateral items, calculating a percentage difference between the two limbs and quantifying the presence of upper limb asymmetry. The Mini-AHA is best used with infants who have already received a diagnosis of unilateral CP. It evaluates use of the more-affected arm/hand during bimanual task performance. The Mini-AHA has more items to evaluate performance of the more-affected hand (20 compared to 17), and uses a 4-point rating scale as compared to a 3-point rating scale on the HAI, which allows for better discrimination of the different ability levels. It is important to recognise that scores from the HAI and Mini-AHA cannot be compared, so the one assessment should be used both at pre-post measurement time points.

One of the major clinical benefits of both the HAI and the Mini-AHA is that they have been developed using Rasch methodology [88,91]. This method uses an iterative process that places both the infant and the items being assessed on the same measurement scale. As well as an infant’s ability to use their more-affected hand during unimanual and bimanual performance being scaled from poor ability to good ability, the items are also ordered this way from items that are easier to perform to items that are harder to perform. This resultant hierarchy provides extremely useful and individualised information for determining action-focused goals and ensuring program specificity.

### 4.3. Using Action-Focused Goals to Determine the Most Appropriate Therapy Approach

In early infancy babies with unilateral CP will have different levels of ability to use their more-affected hand [68]. This is primarily influenced by the timing and type of brain injury [93]. Therefore, it is critical that goal-related, action-focused upper limb therapy programs are designed around a child’s current ability level. Combined with understanding the child and family’s contextual considerations, recognition of a child’s current ability levels allows for selection of the most appropriate model of therapy to target different action-focused goals. In addition, it allows therapists to consider and use different motor learning strategies to optimise skill development and generalisation. In this next section we propose a clinical framework to guide clinicians’ clinical reasoning for the selection of CIMT or bimanual therapy for improving unimanual capacity and bimanual performance in infants with unilateral CP. The specific action-focused goals targeted in each model of therapy will be discussed as well as specific motor learning strategies that may be considered.

## 5. Improving Unimanual Capacity Using Constraint Induced Movement Therapy

**Figure 1:**
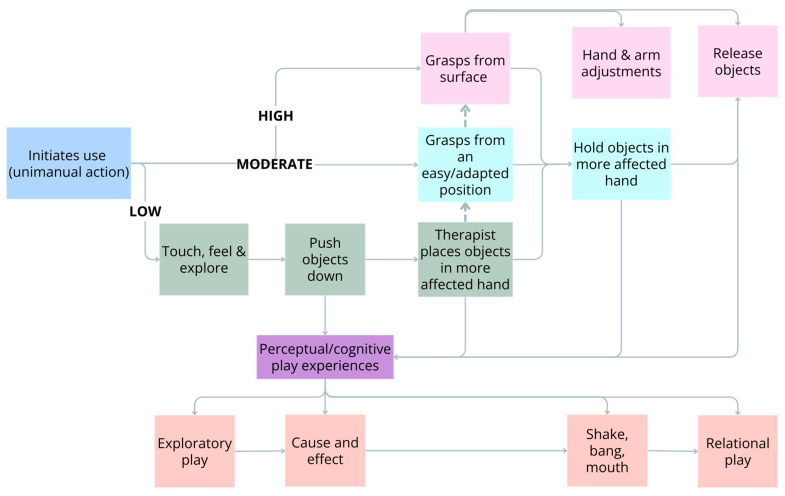
Unimanual action-focused goals targeted using constraint-induced movement therapy.

The best approach to determine the most appropriate model of therapy for an infant with unilateral CP is to start with the end-goal in mind. As previously stated, CIMT provides an opportunity for repetitive practice of unimanual movements and actions [25]. It primarily targets specific unimanual action-focused goals as it is not possible for infants and children to practice bimanual actions when one hand/arm is restrained. If outcomes from the HAI or Mini-AHA identify unimanual goals, e.g., initiates use of the more affected hand/arm, it makes sense that CIMT may be the most appropriate model of therapy to attain these goals (see Figure 1).

### 5.1. CIMT for Infants with Low Ability

Infants with low ability predominantly do not use their more-affected hand/arm at all. It is mostly ignored and held close by their side with their hand fisted. As a result, these infants quickly compensate by only using their less-affected hand to play with toys. They also seem unaware or have an aversion to objects placed in their hand, showing a sensory and behavioural component to their disregard. For these infants’ spontaneous *initiation of use* for an object-related action is a critical first action-focused goal. This aims to increase the infant’s awareness of the more-affected arm/hand to touch, feel or investigate a toy during exploratory play. The spontaneous initiation of use of the more-affected arm/hand should be motivated by the appeal of a toy or the environment, not physical or verbal prompts. The toy affordances should be so motivating that an infant cannot resist trying to touch or feel it. During the early stages of learning it is critical to provide the infant with time to respond to the toy. If they do not respond, providing an auditory cue such as tapping the table with the toy to gain their interest could be used. If they still do not respond it is appropriate to use physical guidance or a prompt, but this should be withdrawn as soon as possible.

Once infants spontaneously initiate use of their more-affected hand/arm toward a toy to touch it, they may then be encouraged to match an appropriate simple action with a desired outcome. For example, knocking over a tower of bricks which promotes understanding of cause and effect. Learning to *push down* with their more-affected hand/arm is also an important unimanual action-focused goal for children with lower ability. For example, pushing down a button of a musical toy to activate it. Learning this action is helpful for later learning how to do a bimanual action-focused goal—*stabilises by weight*/*support*.

Another easy action-focused goal is when the *therapist places a toy in the more-affected hand* to encourage the infant to keep hold of the toy. While this does not require active grasping with the more-affected hand, it requires the infant to maintain hold and facilitates opportunities to perform easy play actions such as waving a bell rattle in the air to make a sound. This allows infants to learn the purpose for holding with their more-affected hand. 

### 5.2. CIMT for Infants with Moderate Ability

For infants with moderate ability who *spontaneously initiate* use of their more-affected hand to reach for and touch toys, a unimanual action-focused goal may concentrate on the infants’ ability to grasp objects. Initially, this may be to *grasp from an easy/adapted position.* This is typically from the therapists’ hand, which allows for the toy to be more stable and appropriately oriented to ensure the task is not too hard or too easy and to help ensure success. If a toy is presented on a surface such as a table or tray, the orientation or lack of stability may make it difficult for the child to grasp effectively, resulting in failure and disinterest. Careful consideration of the affordances of the toy is also essential. For example, when encouraging the infant to grasp from an easy/adapted position, easy to grasp toys should be used. These have features that maximise opportunities for successful grasp such as a thin handle of a rattle or a small maraca. Child initiated grasping also provides opportunities for infants to maintain *hold* of objects in their more affected hand. This allows for self-initiated play actions with the toy such as shaking it, banging it on a table or other surfaces or taking it to the mouth for oral exploration.

### 5.3. CIMT for Infants with High Ability

Once an infant has a more advanced grasping ability with their more-affected hand, unimanual action-focused goals might consider encouraging more difficult actions. These include *grasping from a surface* (such as a tray or table), improving *arm and hand adjustments* when grasping, as well as *releasing* objects. Grasping, holding, and then releasing objects allows for learning about relationships between and within toys. For example, that a brick can be placed in a cup, or a toy coin can be posted into a slot.

Practice should include the opportunity for multiple repetitions of the desired actions which is critical for skill acquisition. Repetition can also be induced by using block practice, whereby you use different toys, but are encouraging the same order of movements/actions. For example, reaching for, grasping, and releasing toys of different shapes and sizes. Modelling and demonstration of the desired goal-related action is also a critical strategy as young children acquire skills by observing and trying to emulate people around them [94].

### 5.4. Considerations for Use of CIMT for Children with Unilateral CP

While CIMT may improve the capacity of an infant’s more-affected upper limb, there are some important considerations when choosing this approach and when to transition away from CIMT. Firstly, the aim of increasing the capacity of the more-affected hand/arm using CIMT is not for this hand to be used for unimanual tasks that are performed when both hands are available (e.g., eating with a spoon or drawing with a crayon). These tasks are typically performed by, and should be performed by, the infant’s less-affected hand as this is quicker, easier and more functional. The aim of building capacity of the more-affected hand/arm is to optimise its ability to be used more successfully as a non-dominant or assisting hand during bimanual task performance. This recognises the seminal work of Lena Krumlinde-Sundholm et al. (2003) when developing the AHA to understand role differentiated behaviour during bimanual task performance, whereby the two hands do not perform the same role [95]. The dominant hand is typically used to do the more manipulative aspects of the task, while the non-dominant hand plays a supporting or assistive role. Even when encouraging unimanual actions during a CIMT program, this should be front of mind.

The second consideration is that improved unimanual capacity does not necessarily lead to improvements in bimanual performance [12]. This is because bimanual actions are more complicated. They involve spatial and temporal coordination of both hands, which requires collaboration of the two hemispheres through the corpus callosum [96]. Skilled bimanual performance also involves perceptual and cognitive processes whereby information from the both the object and the task guides a bimanual response (action), which in turn allows for more precise perception and future actions with both hands [25,66]. This link between cognition/perception and bimanual performance may also explain why some infants with unilateral CP make significant gains in their unimanual capacity following CIMT, but these gains do not translate to improved bimanual skills when both hands are available. As we have previously proposed, learning bimanual skills is best achieved through practice of bimanual tasks as you learn what you practice [25].

## 6. Improving Bimanual Performance Using Bimanual Therapy

As previously stated, we propose that CIMT and bimanual therapy should be viewed as complementary. From this perspective, if CIMT is best used to achieve unimanual action-focused goals, it makes sense that bimanual therapy should be used for infants and young children when learning bimanual action-focused goals [25].

**Figure 2:**
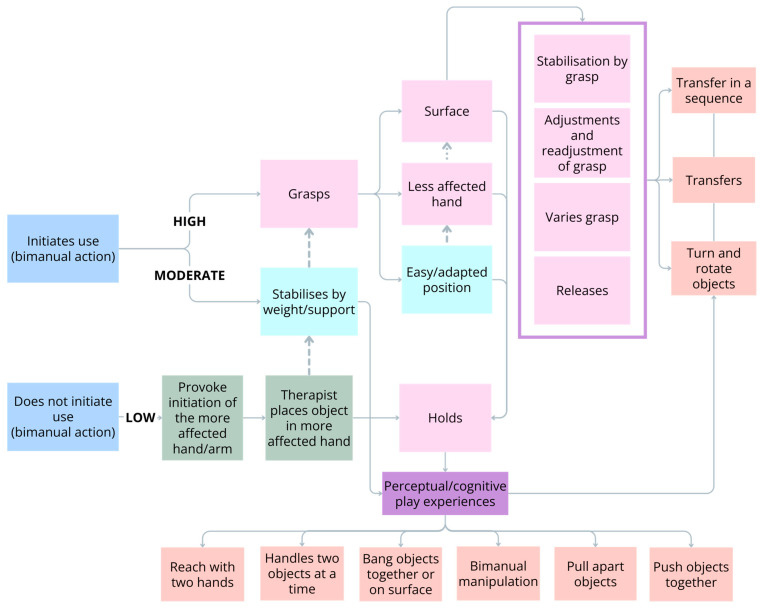
Bimanual action-focused goals targeted using bimanual therapy.

Optimising bimanual performance can be achieved with infants at all levels of ability by supporting the practice of specific action-focused goals that are targeted to an infant’s ability level (see Figure 2). A critical action-focused goal for infants of all ability levels is *initiates use for a bimanual action*. To encourage an infant to use two hands, the task must require the use of two hands. It is the toy affordances that provokes a child to use two hands, not a physical or verbal prompt. The infant must perceive that two hands are needed to be used to complete the task successfully. The age of an infant and cognitive function will likely impact these perceptual experiences.

### 6.1. Bimanual Therapy for Infants with Low Ability

Infants with lower-level ability may not initiate use of their more-affected hand at all when presented with a bimanual toy. For these infants, the therapist may initially need to place toys in the more-affected hand so that toy is *held* and the bimanual task can be completed. While this means that the infant requires the presence of a therapist/caregiver to *grasp* and *hold* the object, the experience facilitates learning of important bimanual play actions such as handling two objects at a time, pulling objects apart or exploring toys during bimanual manipulations. Consistent with CIMT, it is important that the infant should always first be provided with an opportunity to spontaneously initiate use of their more-affected hand/arm. Then, if the infant does not respond to the presentation of the toy you may need to encourage them to initiate by first tapping the toy against their more-affected hand, with or without verbal prompting. These prompts should be withdrawn as soon as possible.

### 6.2. Bimanual Therapy for Infants with Moderate Ability

Once an infant starts to spontaneously initiate use of their more-affected hand, two action-focused goals are important to facilitate bimanual play experiences. The first goal is important when an infant does not use their more-affected hand to grasp toys, but rather uses their fisted hand or their forearm on top of or against an object on a surface or against their body to stabilise it (*stabilisation by weight*/*support*). These infants may have minimal active finger movements, but this does not preclude them from learning to independently pull objects apart or push them together. In most play situations, *stabilisation by weight*/*support* requires the more-affected hand to push down on the object to hold it stably while the less-affected hand completes the bimanual play task, e.g., retrieving a small toy from a container. *Stabilisation by weight*/*support* can be a very effective strategy for stabilising large objects which are difficult to grasp and hold. Careful consideration of the toy affordances and how the play situation is set-up is crucial. To provide an opportunity for an infant to learn when and how to *stabilise by weight*/*support* using a surface, they must first be positioned in sitting with a table or tray. The most suitable toys that provoke *stabilisation by weight*/*support* are those that have parts that need to be pulled apart and have a surface that the infant can place their fist on. However, if the toy is flat or heavy and stable, an infant with unilateral CP will almost always use one hand to play with the toy. The amount of resistance is important as well, as there needs to be just enough resistance so that the activity is not successful unless the child uses their more-affected hand/arm for stabilisation. Because of the cognitive and perceptual requirements for this action, *stabilisation by weight*/*support* can be a challenging strategy for an infant to learn before 12 months of age.

The second important action-focused goal when an infant spontaneously initiates use of their more-affected hand is when the infant can *grasp* with their more-affected hand. In this instance, how much support they need to initiate use and grasp toys with their more-affected hand needs to be considered. During the early stages of practice, we may need to reduce the challenge for the infant to ensure opportunities for success. One way is to use toys that are the right size; for instance, small and thin toys that are easy to grasp. We can also reduce the challenge by holding the toy and offering it in an *easy*/*adapted position* rather than placing it on a table. The aim is to allow an infant to learn the most efficient and effective strategy for *grasping* so that they can use these skills independently in any environment as part of their own repertoire. However, for the infant to learn the reasons for grasping and holding with their more-affected hand, we need to create the right environment by using toys that require the actions of two hands for the play to be successful. Additionally, we need to consider which part of the toy we offer to the more-affected hand to ensure success of the bimanual task.

### 6.3. Bimanual Therapy for Infants with High Ability

Once the infant is successfully grasping toys, a crucial skill for infants with higher ability to learn is to learn how to get a toy into their more-affected hand on their own initiative by *grasping from their less-affected hand*. This action-focused goal can only be taught using bimanual therapy. For many infants with unilateral CP, we know from the item difficulty hierarchy of the HAI and Mini-AHA that this can be the most efficient way to receive toys in the more-affected hand. Importantly, when infants learn this action, more spontaneous use of their more-affected hand is often seen. To learn this action an infant needs first to pick up the desired bimanual toy with their less-affected hand and then transfer the toy into their more-affected hand to *hold*. Strategies such as keeping the toy still in the midline until the more-affected hand also grasps the toy or using physical prompts to guide the more-affected hand towards the toy can be helpful in the early stages of learning. This can be combined with verbal prompts such as “holding” to reinforce the desired action. However, these physical and verbal prompts need to be withdrawn as soon as possible so that the infant initiates the desired action on their own volition. For infants to establish this important goal, it is the repetitive practice of the grasping action in a fun and motivating environment that helps infants learn the desired behaviour.

When an infant can spontaneously and effectively *grasp from their less affected hand* it is important to continue to increase the challenge to evaluate if a more advanced grasping ability can be possible, for example, *grasping from a surface*. To achieve this action-focused goal, first start with easy to grasp toys that are stable and positioned in a way that makes it easier to grasp. However, it is important to increase the challenge as the infants’ abilities improve. This can be achieved by adjusting the toy size, shape, stability or adjusting the environment, e.g., moving the toy further away. It is these strategies that ensures the right challenge and improves the quality of the grasping pattern and motor control. In addition, it is important to think of an appropriate context for motivating grasp from a surface with the more-affected hand. One appropriate situation is when the less-affected hand is occupied, and the infant needs to use their more-affected hand to retrieve a second toy. Another situation may be when a toy is purposefully placed well on the more-affected hand side, making the toy closer to the more-affected hand to grasp. It is important to acknowledge that when an infant has two free hands and the toy is in, or close to midline, they will almost always reach to grasp with their less-affected hand. This is the most efficient and effective strategy to achieve success and not a concern.

As an infant becomes increasingly proficient in *grasping from their less-affected hand* or *grasping from a surface*, action-focused goals that concentrate on how well they are grasping can be addressed. These include their ability to *stabilise objects* well in their more-affected hand, to *adjust* their reaching and grasping according to the conditions of the task, to *readjust their grasp* when necessary as well as the ability to *vary their grasp* according to the toy properties. The ability to use these more complex bimanual actions means that more complex play actions can also be used. For example, transferring a toy from hand to hand in a sequence allows toys to be turned around and explored. Or holding a toy stably in the more-affected hand means that the infant can independently learn to pull apart toys and push them together, which allows also for sequences of play actions such as building magnetic animals. Another natural context to encourage repetitive pulling apart is when an activity has finished and needs to be packed up. Pulling apart the toys and putting them in containers provides significant opportunity for repetitive practice and can often be the most therapeutic part of the activity. This context also provides the opportunity for provoking repetitive release of objects held in the more-affected hand.

## 7. Conclusions and Future Directions

Providing evidence-based upper limb therapy is an essential part of delivering early intervention during a sensitive period of brain development for all infants and young children with unilateral CP. However, key factors to guide clincians about what evidence-based upper limb therapy should be selected for an individual infant/child has not previously been discussed in the literature. Guided by extensive clinical experience and research evidence, this paper provides a best practice framework for planning and implementing individualised, action-focused, goal-directed upper limb therapy programs to optimise outcomes for infants with unilateral CP less than 2 years of age. We propose that there are key considerations for implementing upper limb therapy as well as several factors that influence decision making about what model of upper limb therapy should be selected for an individual infant and their family. These include the family’s goals and preferences, the infants’ current abilities as measured using tools such as the HAI and Mini-AHA, and the generation of specific action-focused goals. In combination, it is this information that determines if CIMT or bimanual therapy would be the most appropriate approach for an infant and their family at a given point in time. The framework also provides a unique clinical perspective for targeting unimanual and bimanual action-focused skills for infants of all ability levels.

While this paper aims to provide a comprehensive clinical framework for implementing upper limb therapy for infants with unilateral CP, we acknowledge that the current research evidence for upper limb therapy approaches for infants with unilateral CP less than 2 years of age remains limited. A systemtatic review of specific therapy approaches in young infants would provide empirical information about the outcome of published research studies. More importantly there is a significant need for additional rigorous, adequately powered studies using well-designed methodologies. These must include comprehensively described therapy protocols to enable evaluation of the specific strategies used in each approach, as well as allow replication of the approach in clinical practice. Longitudinal studies that evaluate the effects of multiple, distributed models of therapy over a longer time period are also required as these therapy approaches are not designed to be applied as a “one-off”.

We also acknowledge that the clincal framework proposed in this paper has not been evaluated in a research study. Further evaluation of which strategies are effective for achieving different action-focused goal for infants at different ability levels and different ages would help clinicians provide the most effective upper limb therapy programs. Additionally, while we are gaining a better understanding of the motor constraints that influence hand use in infancy, a greater understanding of the influence of perception and cognition, particularly on bimanual performance, could identify new strategies to be used when implementing upper limb therapy approaches. Further exploration of how to achieve the best upper limb outcomes for infants with unilateral CP and their families is, and should remain, a critical area for future research.
